# Angiodysplasia of the Gallbladder: An Unknown Risk Factor for Cholecystolithiasis

**DOI:** 10.1155/2020/7192634

**Published:** 2020-08-27

**Authors:** Ivan Švagelj, Mirta Vučko, Mato Hrskanović, Dražen Švagelj

**Affiliations:** ^1^Department of Pathology and Cytology, General County Hospital Vinkovci, 32 100 Vinkovci, Croatia; ^2^Department of Surgery, General County Hospital Orašje, 76 270 Orašje, Bosnia and Herzegovina; ^3^Department of Pathological Anatomy and Forensic Medicine, Faculty of Medicine, University of Osijek, 31 000 Osijek, Croatia

## Abstract

Angiodysplasia is a common type of lesion characterized by malformed submucosal and mucosal blood vessels. Angiodysplasia of the gallbladder is extremely rare, usually an incidental finding, with only two cases reported. Laparoscopic cholecystectomy is a curative treatment for angiodysplasia of the gallbladder. Our report describes a case of angiodysplasia of the gallbladder in a patient who underwent elective laparoscopic cholecystectomy for biliary colic because of gallstones, and a systematic literature review. We surmise that angiodysplasia of the gallbladder could be a risk factor for gallstones in younger female patients.

## 1. Introduction

Vessels are essential, integrative structures of all tissues; therefore, their malformation could be the cause of various pathological conditions. According to the latest WHO classification of tumors of the digestive system, angiodysplasia includes clinically or endoscopically defined entities that are histologically considered to be vascular malformations [1]. This is a type of lesion characterized by malformed submucosal and mucosal blood vessels, which are lined with normal endothelium and surrounded by scant or no smooth muscle but are not related to any hereditary, skin, or systemic disease [2, 3]. The gastrointestinal location is well reported in the literature [4, 5]; however, angiodysplasia of the gallbladder is extremely rare [6] with, to the best of our knowledge, only two cases having been reported [3, 7]. Our report describes a case of angiodysplasia of the gallbladder in a patient who underwent elective laparoscopic cholecystectomy for biliary colic because of gallstones, and a systematic literature review of published cases.

## 2. Case Presentation

A 29-year-old woman was referred to the Department of Surgery of the Orašje County Hospital (Bosnia and Herzegovina) with a history of intermittent colicky right upper quadrant abdominal pain. She had no other significant medical history and had no previous history of gastrointestinal bleeding. Abdominal ultrasonography showed multiple gallstones within the gallbladder, with regular intra- and extrahepatic bile ducts, pancreas, and liver. Routine hematological and biochemical test results were normal. The patient underwent elective laparoscopic cholecystectomy. There were no intraoperative or postoperative complications, and the patient was discharged on the third postoperative day. The patient remained healthy at 6-year follow-up.

**Figure 1:**
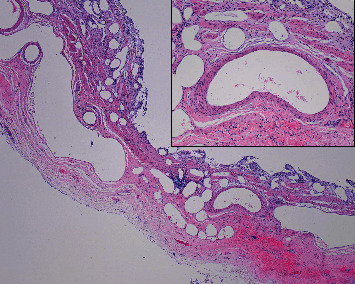
Representative photomicrograph of angiodysplasia of the gallbladder. Low-power magnification shows reduced and wide mucosal folds with cylindrical epithelia of the mucosa almost entirely missing. There was a moderate mixed inflammatory cell infiltrate in the submucosa, and the muscular layer was slightly hypertrophic, which is consistent with chronic cholecystitis. An interesting finding was large, dilated, and malformed arteries and veins in the distorted muscular layer extending into the submucosa and mucosa. (HE; 40x). The inset microphotograph shows a large thick artery from the submucosa of the wall of the gallbladder body (HE; 200x).

**Figure 2:**
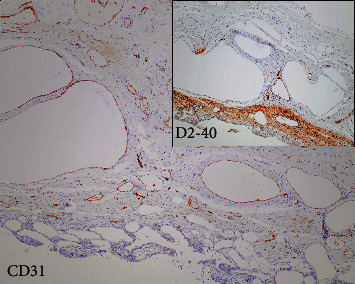
Immunohistochemistry of angiodysplasia. The endothelial lining was confirmed immunohistochemically with positive staining for CD31 and CD34 (not shown), while staining for podoplanin (D2-40) monoclonal antibody was negative (inset microphotograph) (immunohistochemistry; 100x).

Because of the lack of macroscopic pathological alterations, one sample of the gallbladder wall from the body and another from the neck were subjected to microscopic examination, which is a standard routine procedure for gallbladder grossing in our laboratory. Histologically, microscopic slides from the gallbladder body showed that almost the entire cylindrical epithelium of the mucosa was missing, with focal widening of the mucosal folds due to the accumulation of foamy histiocytes and foreign body-type multinucleated giant cells among numerous cholesterol crystal clefts. There was a moderate mixed inflammatory cell infiltrate in the submucosa, and the muscular layer was slightly hypertrophic, which is consistent with chronic cholecystitis. The most unusual feature of the slide from the body of the gallbladder was the presence of large dilated vessels within the muscular layer extending into the submucosa and mucosa (Figure 1). The appearance of angiodysplasia of the gallbladder and its endothelial lining was confirmed immunohistochemically using CD34, CD31, and podoplanin (D2-40) monoclonal antibodies (Figure 2). Of note, previously described histopathological findings were not observed in the slide from the gallbladder neck.

## 3. Discussion

Angiodysplasia is an uncommon lesion that is mainly located in the gastrointestinal tract and may present either no symptoms or gastrointestinal bleeding. This type of lesion occurs at higher frequencies in patients with aortic stenosis, cirrhosis, pulmonary disease, renal failure, and von Willebrand's disease [2]. The most common site is the colon in the lower gastrointestinal tract or the stomach and duodenum in the upper gastrointestinal tract [5, 8]. Except for the gallbladder, which is an extremely rare site of angiodysplasia, cases of the appendix, minor papilla, and proximal bile duct are also described in the literature [9–11].

Most commonly, angiodysplastic lesions are typically seen in elderly patients of both sexes [2]. With respect to angiodysplasia of the gastrointestinal tract, which mainly occurs in patients older than 60 years, angiodysplasia of the gallbladder has been described [3], including our case, in women at the age of 36 and 29 years, respectively (Table 1). This differs from the first published case in which the patient was a 78-year-old male, although he also had angiodysplasia of the gastrointestinal tract and symptoms of gastrointestinal bleeding with a fatal outcome [7].

The pathophysiology of angiodysplasia of the gallbladder (and angiodysplasia generally) remains unclear, although it is likely to be the result of increased contractility of the gallbladder, causing intermittent obstruction of the vessels that penetrate through the muscular wall. One hypothesis is that the occlusion causes focal dilatation and tortuosity of the overlying mucosal vessels, and another is that congestion of the capillaries and precapillary sphincter failure together lead to the formation of arteriovenous collaterals [2, 3]. In our opinion, these ensuing vascular malformations distort the muscular wall, resulting in impaired motility, which is involved in the development of gallstones [12]. Therefore, we suggest that angiodysplasia of the gallbladder could be a risk factor for the development, and may even be the primary cause, of cholelithiasis in younger female patients. Furthermore, the possibility of a causative relationship between angiodysplasia of the gallbladder and gallstones may reveal new insights into the pathophysiology of cholecystolithiasis, although the exceeding rarity [6] of angiodysplasia of the gallbladder could be a limiting factor in further investigations.

Laparoscopic cholecystectomy is curative for the treatment of angiodysplasia of the gallbladder, as evident from our case and the case reported previously [3]. Although our surgeon described the gallbladder mucosa as chronically altered, no technical difficulties were encountered during the operation, and the patient recovered fully with no postoperative complications.

In conclusion, we emphasize the importance of having examined detailed clinical data prior to grossing and tissue processing, to increase the possibility of obtaining an optimal tissue sample and an accurate diagnosis. Hence, the clinical picture of biliary colic and cholecystolithiasis in female patients younger than 40 years should be the “warning sign” for the pathologist to consider the possibility of an angiodysplasia finding.

## Figures and Tables

**Table 1:** Clinical summary of reported cases of angiodysplasia of the gallbladder.

Case (Ref)	Age (years)	Sex	Symptoms	Treatment	Site
1 [7]	78	M	Rectal bleeding	-^∗^	Gallbladder, stomach, duodenum, colon
2 [3]	36	F	Dyspepsia and colicky abdominal pain	Surgery (laparoscopy)	Gallbladder
3 (our case)	29	F	Biliary colic	Surgery (laparoscopy)	Gallbladder

^∗^Fatal outcome.

## Data Availability

Data sharing is not applicable as no new data were created or analyzed in this study.
